# Measuring Cognitive Reserve (CR) – A systematic review of measurement properties of CR questionnaires for the adult population

**DOI:** 10.1371/journal.pone.0219851

**Published:** 2019-08-07

**Authors:** Nadja Kartschmit, Rafael Mikolajczyk, Torsten Schubert, Maria Elena Lacruz

**Affiliations:** 1 Institute for Medical Epidemiology, Biometrics, and Informatics (IMEBI), Medical Faculty of the Martin Luther University Halle-Wittenberg, Halle, Germany; 2 Department of Psychology, Martin-Luther University Halle-Wittenberg, Halle, Germany; University College London Hospitals NHS Foundation Trust, UNITED KINGDOM

## Abstract

**Aim:**

The aim of this systematic review was to summarize and critically appraise the quality of published literature on measurement properties of questionnaires assessing Cognitive Reserve (CR) in adults (>18 years).

**Methods:**

We systematically searched for published studies on MEDLINE, PsycINFO, and Web of Science through August 2018. We evaluated the methodological quality of the included studies and the results on measurement properties based on a consensus-based standard checklist.

**Results:**

The search strategy identified 991 publications, of which 37 were selected evaluating the measurement properties of six different questionnaires. Construct validity of the Cognitive Reserve Index questionnaire was most extensively evaluated, while evaluation of the remaining measurement properties of this questionnaire was scarce. Measurement properties of the Cognitive Reserve Questionnaire and the Cognitive Reserve Scale were assessed more completely. While the Lifetime of Experience Questionnaire seems to be the most thorough instrument, a finale recommendation for one specific questionnaire cannot be drawn, since about half of the measurement properties for each questionnaire were poorly or not assessed at all.

**Conclusions:**

There is a need of high quality methodological studies assessing measurement properties of CR questionnaires, especially regarding content validity, structural validity, and responsiveness.

**Trial registration:**

PROSPERO Registration number CRD42018107766.

## Background

The concept of Cognitive Reserve (CR) emerged from observed discrepancies between age- or pathology-related brain changes and cognitive deficit that one would expect for the particular age or pathology. A high CR is assumed to decrease the susceptibility to clinical manifestations of structural brain changes and is influenced by lifetime experiences [1, 2]. More specifically, several studies reported that higher CR is related to less severe or delayed clinical manifestations in diseases such as Alzheimer’s disease (AD) [3, 4], Parkinson’s disease (PD) [5, 6], traumatic brain injuries [7, 8], and multiple sclerosis (MS) [9–11]. There is evidence that CR is modifiable [12] and that people could change their risk of cognitive decline through performance of mentally and physically stimulating activities. To the extent that existing methods to measure CR are valid and reliable and the causal pathway is considerably strong, CR could be promoted at a population level [13].

However, operationalizing and measuring CR is challenging and various methods are used in current studies to quantify CR. The residual approach treats the variance of cognitive performance that is not explained by demographic variables and brain measures, such as grey matter volume, as current level of CR. The functional imaging approach tries to identify brain networks which possibly underlie CR [14–16]F Another common approach to measure CR is indirectly with sociobehavioral proxy indicators. Commonly used proxy indicators often include education, occupation, physical and leisure activity, and/or premorbid intelligence [13]. While some researchers investigated only a single CR proxy, generally education [17], or included various single proxies in one model in a paralell fashion [18, 19], others combined several proxy indicators and calculated a total score or created latent variable models using for example principle component analysis or structural equation modelling [20–23].

Using a single proxy indicator may not reflect the CR concept appropriately, since CR is a complex construct and determined by various components. Further, empirically determined composite scores and latent variable models, in which the mutual variance among several indicators is used to derive CR score in a specific study [13], lead to very heterogeneous methods and hinder comparability of the results across studies. Hence, attempts have been undertaken to measure CR with standardized questionnaires that include the main proxy indicators of CR [24]. The advantage of such standardized questionnaires is that they include multiple components, provide an a priori defined single score allowing comparison of results from different studies that have used the same questionnaire. Questionnaires may add to a standardization of CR measurements and can be easily distributed, filled in, and analysed in large epidemiological studies.

However, according to our knowledge, no review of CR questionnaires has been performed to date, which is suprising given the severity of emerging symptoms due to age- and disease-related changes. Knowing which questionnaires for assessing CR exist and how they perform will guide researchers in choosing the most appropriate questionnaire for their study. Additionally, appraising their strengths and limitations will guide further research in the development and adaptation of CR questionnaires. The aim of this review is to summarize, critically appraise, and compare the quality of measurement properties of questionnaires aiming to measure CR in diverse adult populations with cognition-related pathologies and in the healthy population.

## Methods

### Literature search

This review was conducted in accordance to PRISMA guidelines [25]. We performed a systematic search in the electronic databases MEDLINE (1946-08/31/2018), Web of Science (1945-08/31/2018), and PsycInfo (1967-08/31/2018). The search was limited to human studies including the terms: [(‘questionnaire’ OR ‘instrument’ OR ‘tool’) AND (‘cognit* OR ‘brain’) AND (‘reserve’ OR ‘reserves’)] OR ‘cognitive reserve’ (MeSH term). The study protocol including the search strategy was uploaded to PROSPERO (www.crd.york.ac.uk/prospero/CRD42018107766).

### Eligibility criteria

We included studies that reported at least one measurement property of a standardized questionnaire for measuring CR, in any of these dimensions: validity, reliability, or responsiveness. We excluded studies that assessed only a subscale of a CR questionnaire. No date and no language restrictions were made. Conference and workshop abstracts were excluded.

We included studies that examined CR in the context of pathology and in healthy populations and did not apply restriction according to a specific disease or health status. Studies conducted in any setting (e.g. samples recruited in hospitals or in the general population) were included without restrictions. We excluded children and adolescents (<18 years old), but no other age restrictions were set.

### Study selection

**Fig 1:**
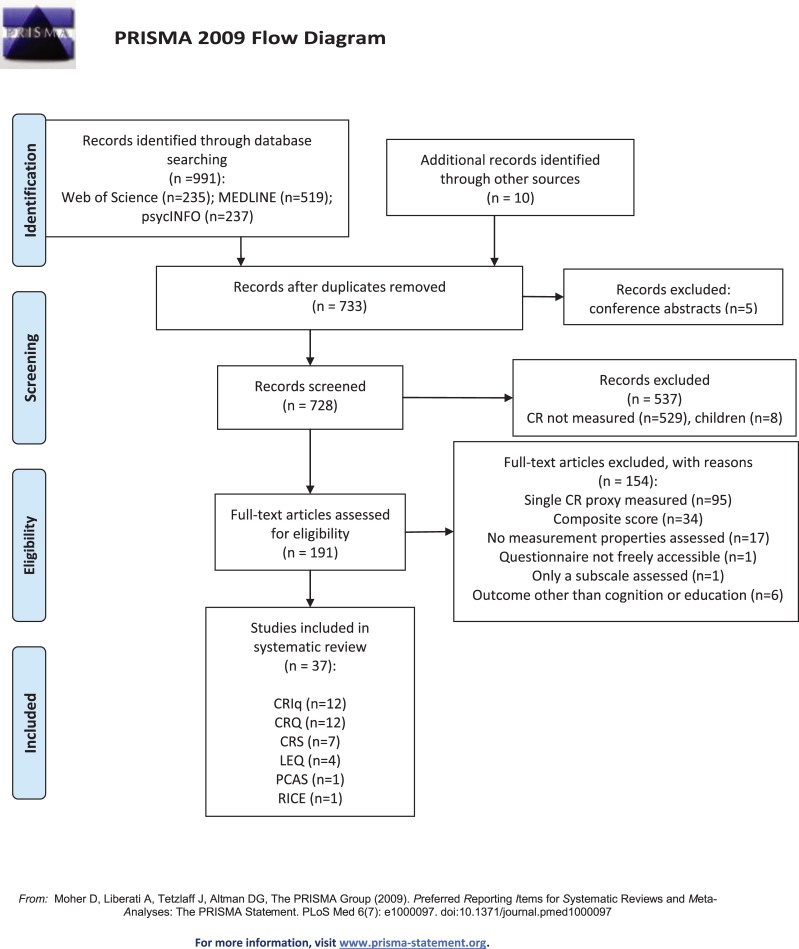
Flowchart article selection.

Two authors (NK and MEL) screened the identified papers and assessed them according to the eligibility criteria. The abstracts of relevant articles were obtained and inspected independently by the two researchers. Discrepancies were resolved by consensus. Justification for excluding studies from the review was documented (see Fig 1).

### Methodological quality assessment and quality criteria

Data extraction templates of the Consensus based Standards for the selection of Measurement Instruments (COSMIN) checklists were used [26, 27]. Data from included studies was independently extracted by the two researchers MEL and NK and transferred into the data extraction templates. A priori agreement on the measurement properties rating was made based on the quality criteria proposed by Terwee et al. [27] and Mokkink et al. [26]. Measurement properties included validity (cross-cultural validity, content validity, construct validity including convergent validity, and structural validity), reliability (internal consistency, reliability, and measurement error), and responsiveness. Measurement properties from each questionnaire could be rated as excellent, good, fair, or poor. We synthesized the evidence regarding measurement properties for each questionnaire taking into account the methodological quality (results from poor methodological studies were not considered), the consistency of the results, and the number of studies.

We adhered to the concept of CR as proposed by Stern and his team [2]. CR refers to “differences in cognitive processes as a function of lifetime intellectual activities or other environmental factors that explain differential susceptibility to functional impairment in the presence of pathology or other neurological insult.” Hence, in order to examine the construct validity of CR questionnaires, ideally 3 components need to be investigated: 1) cognitive status, 2) brain pathology, and 3) CR as a function of intellectual activities and environmental factors. Studies measuring the association between 1) and/or 2) with 3) were included as studies on construct validity. We drew upon the definition of CR proposed by Pettigrew for evaluating construct validity [28]. Thus, we expected higher levels of CR scores, measured by questionnaires, to be associated with

1. better cognitive performance in healthy populations,
2. better cognitive performance *before the onset of cognitive deficits* in populations at risk for cognitive impairment (e.g. patients with MS, PD, HIV); because cognitive symptoms occur later in people with high CR, those people are less likely to be classified as cognitively impaired compared to people with low CR,
3. a more rapid rate of cognitive decline *once cognitive deficits occur*. In cognitively impaired populations, people with higher CR will have greater amounts of neuropathology than people with lower CR given similar clinical symptoms; compared to people with lower CR, people with higher CR will have similar cognitive outcomes in the early stages of cognitive symptom manifestation and worse cognitive outcomes as time from onset of clinical decline goes on: this applies to e.g. people with probable AD where cognitive decline is already present,
4. finally, in studies including brain pathology measures, we expected that the mismatch between brain pathology and cognitive outcomes is higher in people with high CR when compared to people with low CR.

Correlations with other CR proxies (e.g. education, occupation and premorbid intelligence), were used for assessing convergent validity. The definitions of the measurement properties and the quality criteria used for the assessment as described in Rainey et al. [29] and in the COSMIN checklists [26,27] can be seen in Table 1.

**Table 1:** Definition and operationalization of measurement properties (adapted from COSMIN [26,27] and Rainey et al.[29]). CR: Cognitive Reserve.

Term	Definition and operationalization
**Validity**	**The degree to which the CR questionnaire measures the CR construct**
Cross-cultural	The degree to which the translated version of the CR questionnaire adequately reflects the original CR questionnaire. We only considered the methodological quality of the translation process itself. A positive rating was obtained when forward and backward translation, item revision, and a pre-test in the target population of the translated version were performed.
Content	The degree to which the content of the CR questionnaires adequately reflects the CR construct as defined by Stern et al.[2]. A positive rating was obtained when using the theory of Stern [2] as basis, a systematic literature review including expert opinions was performed, different life-stages and dimensions of CR building-activities (e.g. education, occupation, leisure time) were considered, and a pilot study on comprehensiveness and comprehensibility was performed.
Construct	The degree to which the scores of the questionnaire are in line with the CR hypothesis [28]. Studies including measurement of cognitive status with an adequate neuropsychological battery or neuropathology measures were rated as of good methodological quality. Studies including all three dimensions in one model were rated as of excellent methodological quality. A positive rating was obtained when the results were consistent with the CR hypothesis.
Convergent	The degree to which the total score of the questionnaire is related to a common CR proxy (e.g. education, premorbid intelligence). Since common single CR proxies are only one part in building CR and usually describe a concept different from CR, a moderate rather than a high correlation is expected.
Structural	The degree to which the scores of the questionnaire adequately reflect the dimensionality of the CR construct, which can be ascertained with item response theory test, exploratory or confirmatory factor analysis. A positive score is obtained, when the factors explained at least half percent of the variance, comparative fit index was > 0.95, or standardized root mean residual was < 0.08.
**Reliability**	**The degree to which scores of participants without any changes are the same for repeated measurements**
Internal consistency	The degree of interrelationships among items, which is calculated with the Cronbach’s alpha. A positive rating was obtained when there was evidence for adequate structural validity and Cronbach alpha was > 0.70 for each unidimensional scale. Correlations between sub-scales and thus Cronbach’s alpha for the measures is expected to be low, as a CR questionnaire should assess a variety of different proxies and activities.
Reliability	The proportion of total variance due to “real” differences between participants. A positive rating was obtained when intraclass correlation coefficient or weighted Kappa was ≥ 0.70 for dichotomous, nominal, and ordinal scores and Pearson was r ≥ 0.80 for continuous scores, respectively.
Measurement error	The random and systematic error of a score, which is not ascribable to a “real” change in the CR construct. A positive score was obtained when the minimal important change (MIC) was larger than the smallest detectable change (which can be calculated from the standard error of measurement), or when the MIC was outside the limits of agreement.
**Responsiveness**	**The capacity of a CR questionnaire to measure cognitive changes over time. It can be assessed with correlations between cognitive function scores at different time points. For acquiring a positive score, similar requirement as for construct validity were necessary.**

## Results

Thirty seven studies out of 991 screened articles met the inclusion criteria and were included in the review (Fig 1). PRISMA Checklist is presented in S1 File. These 37 studies assessed the following six questionnaires, developed between 2007 and 2017 (Table 2): the Cognitive Reserve Index questionnaire (CRIq, [24]); the Cognitive Reserve Questionnaire (CRQ, [3]); Cognitive Reserve Scale (CRS, [30]), Lifetime of Experiences Questionnaire (LEQ, [31]), Premorbid Cognitive Abilities Scale (PCAS, [32]), and the Retrospective Indigenous Childhood Enrichment (RICE, [33]). The general characteristics of each study on the selected questionnaires are described in Table 3 and more detailed in S2 File. The quality criteria for each questionnaire separately are described below and shown in Table 4 for each study. Table 5 shows the synthesis of the measurement properties per questionnaire.

**Table 2:** Studies included in the systematic review by questionnaire. The study on the development of the questionnaire is marked in bold. SD = standard deviation; Q = quartile; DSM = diagnostic and statistical manual; NINCDS-ADRDA = National Institute of Neurological and Communicative Disorders and Stroke and the Alzheimer's Disease and Related Disorders Association; MMSE = mini mental state examination.

Study	Population	Sampling Method	Total n (% females)	Age in years	Language (country)
**CRIq (Cognitive Reserve Index Questionnaire)**					
**Nucci et al. [24]**	**Participants without evident neurological or psychiatric illness**	**Random selection from the general Italian population**	**588 (55%)**	**Mean 50.21 (SD = 19.62); range: 18–102**	**Italian (Italy)**
Amodio et al. [37]	Participants with cirrhosis without overt hepatic encephalopathy	Outpatients recruited in neurological clinic at university hospital in Padua presenting for the detection of minimal hepatic encephalopathy	82 (27%)	Median 62 (Q1 –Q3 = 54–68)	Italian (Italy)
Arcara et al. [34]	Participants aged 65 or older without psychiatric or neurological illness, MMSE > 24	No information provided	60 (57%)	Mean 73.3 (SD = 7.1), range = 65–98	Italian (Italy)
Ciccarelli et al. [5]	Parkinson disease patients without dementia or other neurological disorders	Random selection of patients at the Center for Medicine of the Aging according to UK Brain Bank criteria	35 (23%)	Mean 76.1 (SD = 7.1)	Italian (Italy)
Fenu et al. [38]	Patients with relapsing remitting Multiple Sclerosis	Recruited at Multiple Sclerosis Centre of University of Cagliari	195 (63%)	Mean 43 (SD = 11.2)	Italian (Italy)
Maiovis et al. [42]	Young adults (18–44 years), middle-aged adults (45–69 years), elderly adults (70–89 years) without major neurologic or psychiatric disorder	Random selection of participants from as many Greek regions as possible	313 young adults (73%), 148 middle-aged adults (54%) and 130 elderly adults (62%)	Young adults mean 28.78 (SD = 7.74), middle-aged mean 56.12 (SD = 7.22), elderly adults mean 75.82 (SD = 4.55)	Greek (Greece)
Maiovis et al. [39]	Patients with behavioural variant frontotemporal dementia and primary progressive aphasia without major psychiatric comorbidities	Recruited from January 2012 to September 2014 in the Neurology Department of a tertiary referral center.	80 (46%)	Mean 67.8 (SD = 8.3)	Greek (Greece)
Milanini et al. [36]	Asymptomatic HIV participants without opportunistic diseases, older than 60 years and without history of neurological disorders, active psychiatric disorders and alcoholism or drug abuse	Recruited from September 2014 to February 2015 during regular outpatient follow-up in three clinical centers (Agostino Gemelli University Hospital, Rome; S. Caterina Novella Hospital, Galatina and Siena University Hospital, Siena)	60 (25%)	Median 66 (range = 62–72)	Italian (Italy)
Mondini et al. [43]	Elderly adults with mild to moderate dementia according to the DSM-V criteria for neuro-cognitive disorders	Recruited from four different geriatric clinics in Northern Italy	86 (75%)	Mean 77.98 (SD = 7.42)	Italian (Italy)
Nunnari et al. [40]	Patients aged 25 or older with Multiple Sclerosis, no current corticosteroid use and other neurological or psychiatric illnesses	Recruited consecutively from June 2014 to February 2015 at the IRCCS Center Neurolesi Bonino Pulejo of Messina	66 (65%)	Mean 39.5 (SD = 9.7)	Italian (Italy)
Puccioni & Valessi [35]	Older adults without dementia and younger controls (CR assessed in older adults only)	No information provided	17 older adults (47%), 18 younger adults (50%)	Older adults mean 73 (range = 69–79), younger adults mean 24 (range = 18–34)	Italian (Italy)
Volpi et al. [41]	Patients with subjective cognitive impairment (SCI) and mild cognitive impairment (MCI)	Patients recruited at the Memory Unit of the Neurological Clinic, University of Pisa between January 2010 to July 2013	93 SCI patients (45%), 93 MCI patients (52%)	SCI patients mean 72.8 (SD = 5.8), MCI patients mean 75.1 (SD = 4.8)	Italian (Italy)
**CRQ (Cognitive Reserve Questionnaire)**					
**Rami et al. [3]**	**Mild-Alzheimer disease patients (according to NINCDS-ADRDA criteria) and cognitively healthy controls aged 65 years or older**	**Patients recruited at the unit for Alzheimer and other cognitive impairments at Hospital of Barcelona. No information on recruitment of controls**	**55 controls (51%), 53 mild Alzheimer disease patients (58%)**	**Control mean 73.8 (SD = 6.0), patients mean 77.7 (SD = 5.0)**	**Spanish (Spain)**
Bartres-Faz et al. [45]	Participants without neurological or psychiatric medical diagnosis	Participants from the Barcelona Brain Health Initiative (prospective longitudinal cohort) meeting the study's inclusion criteria	1081 (63%)	Mean 52.0 (SD = 7.1); range = 40–65	Spanish (Spain)
Ferreira [47]	Healthy middle-aged adults without psychiatric or neurologic disorders and MMSE ≥26	Recruited from the GENIC-database (Group of Neuropsychological Studies of the Canary Islands)	82 (51%)	Mean 45.1 (SD = 3.9)	Spanish (Spain)
Harris et al. [54]	Participants with mild cognitive impairment, Alzheimer’s disease and no cognitive impairment	Participants from the Argentina Alzheimer’s Disease Neuroimaging Initiative (ADNI) database recruited at the Neurological Institute of Investigation meeting the study's inclusion criteria	33 (8 normal cognitive aging (50%), 23 MCI (48%), 2 AD (0%))	Normal aging mean 60.75 (SD = 6.67), MIC mean 65.88 (SD = 7.10), AD mean 81 (SD = 4.24)	Spanish (Argentina)
López-Higes et al. [50]	Participants aged 60 years and older with MMSE ≥26	Recruited from different nursing homes and day care centers in Madrid	83 (53%)	Mean 64.8 (SD = 4.3)	Spanish (Spain)
López-Higes et al. [57]	Participants with MMSE ≥24 at baseline	Recruited from the Center for Cognitive Impairment Prevention (Public Health Institute, Madrid City Council)	81 (68%)	Cognitively intact participants’ mean 70.9 (SD = 4.2); participants with subjective cognitive decline mean 71.4 (SD = 4.8)	Spanish (Spain)
López-Higes et al. [51]	Participants aged 60 years and older with MMSE > 26	Recruited from different nursing homes and day care centers in Madrid	83 (53%)	Mean 64.8 (SD = 4.3), range = 60–75	Spanish (Spain)
Pedrero-Pérez et al. [44]	Patients under treatment for substance addiction without cognitive impairment	Recruited consecutively in public health care center for treatment of substance addiction	57 (30%)	Mean 39 (SD = 13) in men; mean 44 (SD = 12) in women	Spanish (Spain)
Sobral et al. [56]	Outpatients with probable Alzheimer disease according to DSM-IV criteria	Convenience sample recruited at the psychogeriatric service of a psychiatric hospital	75 (73%)	Mean 80.2 (SD = 5.64); range = 61–92	Portuguese (Portugal)
Sobral et al [53]	Outpatients with probable Alzheimer disease according to DSM-IV criteria	Convenience sample recruited at the psychogeriatric service of a psychiatric hospital	75 (73%)	Mean 80.2 (SD = 5.64); range = 61–92	Portuguese (Portugal)
Vasquez-Amezquita [49]	Adults aged 60 years or older, right-handed and without psychiatric or neurological diseases	Convenience sample recruited from day care center in Ibagué (Colombia)	30 (87%)	Mean 70.0 (SD = 6.6)	Spanish (Colombia)
Wikee & Martella [52]	Healthy, elderly adults (> 65 years); group 1: adults with arthrosis performing mild to moderate physical activity; group 2: adults performing physical activity at least 2 times per week; group 3: physically inactive adults	Recruited at a sports center and the community Pudahuel, Santiago de Chile	60 (20 per group, group 1: 85%, group 2: 85%, group 3: 90%)	Group 1 mean 71 (SD = 4.4), group 2 mean 69 (SD = 3.3), group 3 mean 71 (SD = 6.4)	Spanish (Chile)
**CRS (Cognitive Reserve Scale)**					
**León I. et al. [30]**	**Adults and elderly without psychiatric or neurologic disorder or CVD, no drug consumption or trauma**	**Recruited at the virtual campuses of universities, community centres and neighborhood societies of Almería.**	**95 (75 younger adults (75%), 20 older adults (55%))**	**Younger adults mean 23.55 (SD = 2.52), older adults mean 65.1 (SD = 5.73)**	**Spanish (Spain)**
Altieri et al [58]	Participants without psychiatric or neurological illness, drug or alcohol abuse, MMSE > 23.8	Convenience sample recruited at universities, churches, gyms, and community centers	547 (50%)	Mean 49.2 (SD = 20.2); range 18–89	Italian (Italy)
Cancino et al. [59]	Older participants without neurological disease or depression	Convenience sample (no further information provided)	206 (77%)	Mean 69 (SD = 0.5)	Spanish (Chile)
León et al. [60]	Adults (aged 36–64 years) and elderly adults (≥65 years) without history of psychiatric or neurological illness, drug consumption or head injury, MMSE >27	Recruited from social clubs, social centers, entertainment centers and the University of Almeria	117 (87 adults (62%) and 30 elderly adults (73%))	Adults mean 49 (SD = 0.8), elderly adults mean 73 (SD = 1.1)	Spanish (Spain)
León et al. [61]	Healthy individuals older than 65 years	No information provided	30 (73%)	Mean 72.9 (SD = 6.0)	Spanish (Spain)
León-Estrada et al. [63]	Adults (aged 36–64 years) and elderly adults (≥65 years) without history of psychiatric or neurological illness, drug consumption or head injury, MMSE >27	Recruited from social clubs, social centers, entertainment centers and the University of Almeria.	172 (110 adults (60%), 62 elderly adults (65%))	Adults mean 48.54 (SD = 7.29), elderly adults mean70.52 (SD = 5.61)	Spanish (Spain)
Roldan-Tapia et al. [62]	Adults (aged 36–64 years) and elderly adults (≥65 years) without history of psychiatric or neurological illness, drug consumption or head injury, MMSE >27	Recruited from social clubs, entertainment centers, and the University of Almeria’s Center for Adult Education.	140 (98 adults (65%), 42 elderly adults (69%))	Adults mean 49.15 (SD = 7.18). Elderly adults mean 71.88 (SD = 5.62)	Spanish (Spain)
**LEQ (Lifetime of Experience Questionnaire)**					
**Valenzuela et al. [31]**	**Healthy participants aged 60 years or older**	**Participants from the control arm of the Sydney Stroke Study**	**79 (57%)**	**Mean 70.9, range = 58–93**	**English (Australia)**
Hindle et al. [64]	Parkinson disease patients with MMSE ≥26 above 60 years and Hoehn-Yahr scale stage 1 to 3	Recruited through Movement Disorder Clinics in District Hospitals run by Geriatricians or Neurologists with specific expertise and training in the assessment and management of Parkinson disease (part of the Bilingualism as a protective factor in Age-related Neurodegenerative Conditions (BANC) study)	69 (28%)	Mean 73.1 (SD = 6.7)	English (UK)
Lavrencic et al. [65]	Right-handed older adults without uncontrolled hypertension, recent history of cancer or any neurological or psychiatric disorder	Recruited from Magill and surrounding areas, South Australia	115 (62%)	Mean 68.5 (SD = 5.9); range = 60–85	English (Australia)
Opdebeeck et al. [66]	Healthy participants aged over 60 years	Recruited from Agewell centers, over 50s clubs, church groups, active retirement groups, and flyers advertising the study using purposive snowball sampling	236 (62%)	Mean 71 (SD = 7.7), range = 60–92	English (UK and Republic of Ireland)
**PCAS (Premorbid Cognitive Abilities Scale)**					
**Apolinario et al. [32]**	**Participants 60 years or older: normal cognitive aging, mild cognitive impairment (MCI) or mild dementia according to the Clinical Dementia Rating**	**Recruited in a geriatric memory clinic at the University of Sao Paulo (Brazil)**	**132 (72 normal cognitive aging (81%); 33 MCI (76%) and 27 mild dementia (74%))**	**Normal aging mean 73.0 (SD = 7.8), MCI mean 73.9 (SD = 5.7), mild dementia mean 74.7 (SD = 7.2)**	**Portuguese (Brazil)**
**RICE (Retrospective Indigenous Childhood Enrichment scale)**					
**Minogue et al. [33]**	**Aboriginal Australian people without cognitive impairment**	**Study 1: population-based sample of Australian Aboriginal people aged 60 years and older living in in New South Wales who participated in Koori Growing Old Well Study; Study 2: convenience sample of participants aged 50 years and over living in Sydney who were identified as Aboriginal Australian**	**Study 1: 294 (60%); Study 2: 38 (66%)**	**Study 1 mean: 66.61 (SD = 6.3), Study 2 mean 70.15 (SD = 8.38).**	**English (Australia)**

**Table 3:** General characteristics of the studied CR questionnaires. CRIq = Cognitive Reserve Index Questionnaire, CRQ = Cognitive Reserve Questionnaire, CRS = Cognitive Reserve Scale, LEQ = Lifetime of Experience Questionnaire, RICE = Retrospective Indigenous Childhood Enrichment scale, PCAS = Premorbid Cognitive Abilities Scale.

Questionnaire	Life-span	Target population	Dimensions/subscales	Mode of administration	Number of items, response options	Administration time	Scoring	Available in
CRIq	Unrestricted	General adult population	Education, working activity, leisure time	Self-report or relative-report (semi-structured interview)	24 items, numerical scale (number of years) and dichotomous answers	15 minutes	3 sub-scores for the dimensions, 1 total score with 5 levels according to sum of total score (low to high CR)	Italian, Greek, English, French, German, Spanish, Portuguese, Catalan, Czech, Dutch, Latvian
CRQ	Unrestricted	General adult population	Education, parent's education, leisure time, bilingualism	Self-report or relative-report	8 items, 3 to 6-point Likert scale	2 min.	Score ranging from 0–25	Spanish, Portuguese
CRS	3 stages (18–35 years, 36–64 years, over the age of 65)	General adult population	Daily activities, training information, hobbies, and social life	Self-report	24 items, 5-point Likert scale	15 min.	Score ranging from 0–96	Spanish, Italian, English
LEQ	3 stages (between 13 and 30 years, from 30 to 65 years and from 65 years)	General adult population	Specific (education, occupation) and non-specific mental activity (leisure time) for each life-span	Self-report	42 items, 6-point Likert scale and open questions	30 min.	3 sub-scores for life-spans, 1 total score with each dimensions contributing equally	English
PCAS	Premorbid period with 10-year time frame	Low-educated population with dementia	Education, occupation, reading, writing, and calculation abilities, use of technology, abilities to search for information, reading habits	Relative-report	19 items, 2-point to 6-point Likert Scale	Not reported	Score ranging from 0 to 30	Portuguese, English
RICE	Up to 15 years of age inclusively	Aboriginal Australians	Traditional, community, physical activity, reading and playing games	Self-report	21 items, 5-point Likert scale	Not reported	Score ranging from 19 to 81	English

**Table 4:** Risk of bias in each study on measurements aspects by questionnaire (the terms denote the quality of measurement, not the result of the measurement).

Study	Cross‐cultural validity *	Content validity	Construct validity	Convergent validity	Structural validity	Internal consistency	Reliability (test-retest)	Measurement error	Responsiveness
**CRIq**									
**Nucci et al. [24]**		fair	fair	good		poor			
Amodio et al. [37]			excellent						
Arcara et al. [34]			fair						
Ciccarelli et al. [5]			good						
Fenu et al. [38]			excellent						
Maiovis et al. [42]	fair		poor						
Maiovis et al. [39]			excellent						
Milanini et al. [36]			good						
Mondini et al. [43]									poor
Nunnari et al. [40]			excellent						
Puccioni & Valessi [35]			fair						
Volpi et al. [41]			good						
**CRQ**									
**Rami et al. [3]**		poor	good						
Bartres-Faz et al. [45]			good						
Ferreira [47]			good						
Harris et al. [54]			good						
López-Higes et al. [50]			poor						
López-Higes et al. [57]									fair
López-Higes et al. [51]			fair						
Pedrero-Perez et al. [44]			good	fair	good	good			
Sobral et al. [56]					good	good			
Sobral et al [53]	fair		good						
Vasquez-Amezquita et al. [49]			poor						
Wikee & Martella [52]			poor						
**CRS**									
**León I. et al. [30]**		good	poor	fair		poor			
Altieri et al [58]	good		good	good		poor	good		
Cancino et al. [59]			good						
León et al. [60]			good	good		poor			
León et al. [61]			poor						
León-Estrada et al. [63]					fair	poor	poor	fair	
Roldan-Tapia et al. [62]			good						
**LEQ**									
**Valenzuela et al. [31]**		fair		good	poor	poor	good		good
Hindle et al. [64]			good						
Lavrencic et al. [65]			fair						
Opdebeeck et al.[66]			good						
**PCAS**									
**Apolinario et al. [32]**		fair	fair	good	good	good	good		
**RICE**									
**Minogue et al. [33]**		good		good	good	good	good		

* Only the translation process was evaluated.

**Table 5:** Synthesis of measurement properties per questionnaire (terms denote quality of evidence, not the content). + or - moderate evidence positive/negative results, +/- conflicting evidence, ? Only poor methodological studies or not all information for proper assessment reported, N/A information not available Synthesis derived from a single study.

Questionnaire	Content validity	Structural validity	Internal consistency	Reliability	Measurement error	Hypotheses testing for construct validity	Convergent validity	Responsiveness
CRIq	+	N/A	?	N/A	N/A	+	+	?
CRQ	?	+	+	N/A	N/A	+	+	-
CRS	+	+	-	+	?	+/-	+/- (3 studies)	N/A
LEQ	+	?	-	+	N/A	+/-	+	+
PCAS	+	+	+	+	N/A	+	+	N/A
RICE	+	?	+	+	N/A	N/A	+	N/A

### CRIq

There was fair evidence for the content validity of the Italian version of CRIq. Nucci et al. [24] reported to use the CR theory of Stern [2] as conceptual framework for the development of the questionnaire. No pilot study was conducted to assess comprehensibility and comprehensiveness of the items in the target population. Information on the target population and context use was provided [24].

Whereas there were less studies examining construct validity in the healthy population, a series of studies supports the construct validity in diseased populations. In a healthy population, Arcara et al. [34] found that CRIq working activity score was significantly associated with informal math use in daily life (t = 3.4, p = 0.001), but no other CRIq scores were significantly related to any of the nine different math tests in a healthy population (no effect estimates and no statistical significance reported). In healthy older adults, Puccioni [35] found that higher CRIq score was associated with decreased response time difference between congruent-incongruent and incongruent-incongruent sequences of a spatial Stroop task (r = -0.51, p = 0.036), which is assumed to be a measure of cognitive control resources of a person. In patients, Ciccarelli et al. [5] found that higher CRIq scores were independently associated with a better performance on Word Fluency (β = 0.40, p = 0.04) and Digit Span (backwards) (β = 0.38, p = 0.02) in PD patients without dementia. However, long-term and working memory as well as reasoning were not associated with CR (no effect estimates or significance values reported), which could mean that the positive effect of CR in PD patients is domain specific rather than universal. In line with the definition of CR, Milanini et al. [36] found that the total CRIq score was associated with a lower risk of cognitive impairment, defined as test performance below an adjusted cut-off in at least 2 cognitive measures (OR = 0.94; 95% CI = 0.91–0.97) in asymptomatic patients with HIV without history of neurological disorders. Additionally, total CRIq score was associated with higher global and single z-scores of cognitive performance, i.e. better cognitive function (β = 0.39, p = 0.002).

Most studies including both, brain pathology and cognitive function measures, support the hypothesis that CRIq scores can account for the mismatch between cognitive performance and pathology. Amodio et al. [37] found that the discrepancy between cognitive and neurophysiological assessment in non-overt Hepatic Encephalopathy (expressed as cognitive performance normalized by EEG speed) was found to be positively correlated with the total CRIq score (r = 0.36, p<0.01). In Multiple Sclerosis (MS) patients, Fenu et al. [38] showed a significant association between the Brief International Cognitive Assessment for Multiple Sclerosis (BICAMS) and the interaction between CRIq scores and cortical gray matter volume, adjusted for age and disability (p = 0.004, no effect estimates reported). In patients with frontotemporal dementia, Maiovis et al. [39] found that higher total CRIq, CRIq -Leisure Time, and CRIq-Education scores were associated with lower regional cerebral blood flow (rCBF) in the bilateral frontal and left temporal cortex (e.g. association between the CRIq total score and the left frontal rCBF for the behavioral variant frontotemporal dementia group (F = 3.7, p = 0.008) and for the primary progressive aphasia group (F = 4.502, p = 0.004). Nunnari et al. [40] found that CRIq total score was associated with some cognitive measures (e.g. Selective Reminding Test Consistent Long Term Retrieval: β = 0.24, p = 0.04, Symbol Digit Modalities Test: β = 0.29, p<0.001, Paced Auditory Serial Addition Test 3: β = 0.32, p<0.001) in patients with MS. However, the interaction between brain pathology (measured with normalized cortical volume) and CRIq scores did not contribute significantly to the explanation of cognitive measures (β ranging from 0.0006, p = 0.75 to 0.01, p = 0.12). Volpi [41] aimed to identify factors discriminating between participants with subjective cognitive impairment (SCI) and mild cognitive impairment (MCI) and found that higher total CRIq score was associated with slightly lower probability of having MCI (OR = 0.971, 95%CI = 0.948–0.995).

Regarding convergent validity, Nucci et al. [24] found the vocabulary test of the Wechsler Adult Intelligence Scale (WAIS) and the short intelligence test (TIB) to be moderately associated with the total CRIq score (r = 0. 42 and r = -0.45, respectively) in a healthy population.

There was poor evidence for internal consistency, since structural validity has not been assessed in any of the studies. Nucci et al. [24] reported a good Cronbach alpha for the CRIq -Leisure Time subscore (α = 0.73, 95% CI = 0.70–0.76) and a poor alpha for the total scale (α = 0.62, 95% CI = 0.56–0.97). No alpha values were reported for the CRIq-subscores Education and Working Activity. In the study by Nucci et al. [24], the total score correlated with all three sub-scores (r = 0.8 for education and working activity, and r = 0.7 for leisure time) and correlations between sub-scores were low (e.g. education and leisure time r = 0.3), and consistent results were found for the Greek version (e.g. education and leisure time r = 0.36 and r = 0.36 respectively) [42].

No information regarding test-retest reliability or measurement error for the CRIq was found.

With only one study, there was poor evidence on the responsiveness of the CRIq to interventions. In patients with mild to moderate dementia, Mondini et al. [43] found that the global cognitive status, measured with the Mini Mental State Examination (MMSE) of participants with lower total CRIq score improved more after cognitive treatment, than in participants with higher total CRIq score (t = 3.958, p<0.001).

### CRQ

Construct validity of the CRQ has been validated in healthy populations and in patients with probable AD. One further study examined patients under treatment for substance addiction without cognitive impairment [44]. In the healthy general population, Bartres-Faz et al. [45] found that the CRQ was associated with self-reported cognitive function (β = 0.008, p<0.005), and this association was mediated by purpose in life, measured with the PiL subscale of the Spanish version of Ryff’s Well-Being Scale [46] (95% bootstrap CI = 0.03–0.11) and sense of coherence (95% bootstrap CI = 0.03–0.08). Also in healthy participants, Ferreira et al. [47] reported that CRQ mediated the effect of thinning in the left middle-temporal gyrus and the left entorhinal cortex on the Color Trails Test-2 (averaged attenuation effect = 52%). However, no mediation effect was found for block design, measured with the Spanish version of the California Verbal Learning Test (TAVEC)[48] (β = 0.213, p = 0.091) and for the Judgment of Line Orientation Test (β = −0.081, p = 0.602). Vasquez-Amezquita [49] reported that besides education, no item of the CRQ was associated with a neuropsychological battery (Neuropsi) in a stepwise-linear regression model including healthy participants (information on correlation with the total CRQ score was not provided).

Some items of the CRQ were correlated with cognitive measures in a study by López-Higes et al. [50], e.g. for the Boston Naming Test spontaneous answering and schooling (p<0.003). However, no effect size estimates and no correlations with the total score were provided. In another study by López-Higes [51] in the same population, higher CRQ score was associated with decreased diversity in the Boston Naming Test (BNT) and in verb and sentence comprehension with two propositions (e.g. β = -0.273, p = 0.013 for the BTN). Possibly due to homogeneity of the CRQ scores in the sample, Wikee & Martella [52] were not able to show any associations between CRQ scores and functionality of the attentional networks when comparing three groups of cognitively healthy participants with different physical activity levels (neither effect estimates nor statistical significance were reported).

Four studies reported construct validity of CRQ in patients with AD and one in substance-addicted patients under treatment. Pedrero-Perez et al. [44] showed that higher CRQ scores were associated with better cognitive function in substance-addict patients under treatment without cognitive impairment (r = 0.38, p<0.01). Rami et al. [3] found associations between the CRQ and Tests of Attentional Performance (r = 0.62 p<0.001 in healthy adults and r = 0.75 p<0.001 in adults with AD). In patients with probable AD, Sobral et al. [53] reported a relationship between the Clinical Dementia Rating and the CRQ (p = 0.02, no effect estimate provided). A comparative study of participants with different degrees of cognitive impairment showed that CRQ was positively associated with underlying brain pathology, measured with amyloid deposition (Aβ1–42) (ρ = 0.42, p<0.05) [54]. Regarding convergent validity, Pedrero-Pérez et al. [44] observed a correlation between the CRQ and premorbid intelligence based on sociodemographic data as proposed by Bilbao and Seisdedos [55] (r = 0.65, p<0,001).

There was good evidence on the structural validity and internal consistency of the CRQ. Two studies performed exploratory factor analysis for the assessment of the dimensionality of the CRQ, one using the Portuguese version in a population of probable AD patients [56] and the other using the Spanish version in a population of patients under addiction treatment [44]. In both cases, the factor analysis and by root mean square residual supported the unidimensionality of the CRQ, which was a good indicator of the quality of the adjustment (comparative fit index = 0.99 and 0.96 respectively) (RMSR = 0.05 and RMSR = 0.07 respectively) [44,56]. The Spanish version showed excellent internal consistency with a Cronbach’s alpha of 0.96 [44] and the Portuguese version of the CRQ showed good internal consistency with a Cronbach’s alpha of 0.80 [56].

No information regarding reliability and measurement error was found.

Regarding responsiveness, López-Higes et al. [57] reported that, after cognitive training, CRQ was a significant predictor for improved MMSE for participants with cognitive impairment (partial ŋ^2^ = 0.105, p = 0.0025), but not for cognitively intact older adults (partial ŋ^2^ = 0.030, p>0.005).

### CRS

There was good evidence for the content validity of the CRS [30]. Items of the CRS were determined through systematic literature review and expert opinions. A pilot study was conducted which resulted in the elimination of some items and reduction of the life stages. However, no information on comprehensiveness and comprehensibility in the target population was given. Although education and occupation are the most common CR proxies that are reported in the literature, these items are not included in the CRS score, limiting the content validity. However, the CRS allows for measuring CR besides education and to capture differences in groups with similar educational background.

There was inconsistent evidence on the construct validity of the CRS in healthy participants when taking into account that the methodological quality was overall good, but about half of the studies could not find expected significant associations between cognitive outcomes and the CRS. Altieri et al. [58] found weak positive associations between CRS scores and global cognitive functioning (MMSE, r = 0.26, p<0.001 and Montreal Cognitive Assessment, r = 0.28, p<0.001), long-term verbal memory (immediate recall Rey Auditory Verbal Learning Test (RAVLT), r = 0.25, p<0.001 and delayed recall RAVLT, r = 0.25, p<0.001) and visuo-spatial constructional abilities (Constructional Apraxia Test, r = 0.19, p<0.001). Cancino et al. [59] found an association of the CRS with the Addenbrooke's Cognitive Examination-Revised (β = 0.223, p = 0.005). León et al. [60] found significant association between the CRS and several cognitive measures (e.g. Verbal Learning Spanish–Complutense Test last trial: r = 0.24, p = 0.009, short-term memory: r = 0.29, p = 0.002, and long-term memory: r = 0.22, p = 0.018). However, no significant correlations were found with any test of processing speed, attention, and working memory (e.g. Digit Span backward r = 0.077, p = 0.409, Stroop word-colour score r = 0.135, p = 0.147). This is in line with the results of another study performed by León et al. [61], where the CRS score was related to Rey-Osterrieth Complex Figure short-term-recall (partial ŋ^2^ = 0.32 p = 0.002) and long-term-recall (partial ŋ^2^ = 0.3, p = 0.003), but not with attention (p = 0.287, no effect size reported) and working memory (p = 0.47, no effect size reported). Roldan-Tapia et al. [62] found that inhibition was significantly associated with CRS (β = 0.49; p = 0.007; r = 0.23), but not with other executive functions. No significant correlation was found between the CRS and perception of cognitive status (r = -0.16, no significance value reported) in a study performed by León et al. [30]. There was no study examining the construct validity of the CRS in diseased populations.

Regarding convergent validity, León et al. [30] found that higher CRS score was negatively correlated with premorbid intelligence measured with Bilbaos & Seisdedos sociodemographic formula (r = -0.31). In a further study by León et al. [60], a correlation between the CRS and years of education (partial ŋ^2^ = 0.07, p = 0.004) was found, but not with premorbid intelligence as measured with a Vocabulary subtest (r = 0.09, p = 0.33), nor occupation (F_2,116_ = 0.11, p = 0.898). In the Italian version, Altieri et al [58] found associations between the CRS and years of education and occupation (r = 0.33 and ρ = 0.16, respectively).

There was moderate evidence for the structural validity and four studies on internal consistency, but of low quality. For the total scale, León et al. [30,60] reported adequate Cronbach’s alphas of 0.81 and 0.77, respectively, but structural validity was not assessed beforehand. Since unidimensionality was not examined beforehand, the Cronbach’s alpha values are difficult to interpret. León-Estrada et al. [63] performed exploratory factor analysis for assessing structural validity. They provided evidence of the bidimensionality of the CRS (a general and four sub-scales, namely activities of daily living, education, hobbies and social life) and of a good indicator of the quality of the adjustment (comparative fit index = 0.9) and by root mean square residual (0.04) and [63] reported a Cronbach’s alpha of 0.8 for the total scale. However, Cronbach alphas for the four sub-scales were not provided. Altieri et al. [58] reported a Cronbach’s alpha of 0.73 for the total scale. For the different life-stages, alpha values ranged from 0.738 and 0.747. However, structural validity was not assessed beforehand in this study.

Altieri et al. [58] provided good evidence of the test-retest reliability of the CRS in a subsample of 15 participants, who were comparable to the whole sample according to demographic characteristics. Scores of the total CRS showed to be reliable over 4-weeks (r = 0.983, p<0.001). León-Estrada et al. [63] provided poor evidence for test-retest reliability and moderate evidence for measurement error of the CRS. The intraclass correlation (ICC) was calculated using the three different life-stages of the CRS, which does not provide an adequate method for assessing reliability. ICC ranged from 0.50 (95% CI = 0.27–0.66) to 0.92 (95% CI = 0.87–0.95) for the same items in the different life stages. The Standard Error of Measurement (SEM = 4.96) was calculated, but no information on the minimal important change was provided. No information on responsiveness was found.

### LEQ

There was fair evidence of the content validity of the LEQ [31]. The authors of LEQ report to have derived the items based on literature research, but it is unclear how this research was performed. The authors indicate that the LEQ has excellent face validity, but do not provide information about relevance, comprehensibility, and comprehensiveness of the items according to experts and the target population.

The studies on construct validity were of fair to good methodological quality, but the results indicate rather poor evidence of construct validity. In PD patients without cognitive impairment indicated by a MMSE score of 26 or higher, Hindle et al. [64] found that the LEQ total score was positively associated with executive function. LEQ mid-life score was positively associated with mental generativity, assessed through verbal fluency (word generation after letter- and category cues; r = 0.25, p<0.05), design fluency (novel design generation by connecting dot arrays with 4 straight lines; r = 0.26, p<0.05), tasks and set shifting and switching (Test of Everyday Attention Elevator Counting; r = 0.25, p<0.05). However, after correction for multiple comparisons, there was no significant difference in executive functions between participants with LEQ scores either higher or lower than the mean LEQ score (e.g. mean verbal fluency total score 44.35 (SD = 9.65) for participants with LEQ score higher than the mean and 39.30 (SD = 13.23) for participants with LEQ score lower than the mean, p = 0.142). In a healthy population, Lavrencic et al. [65] found that the LEQ did not predict performance on any tests of The Awareness of Social Inference Test (e.g. Emotion Evaluation Test: β = -0.097, p = 0.325, Social Inference–Minimal: β = -0.004, p = 0.972, Social Inference–Enriched: β = -0.016, p = 0.878). In contrast, Opdebeeck et al. [66] demonstrated that higher LEQ score was associated with better performance in delayed recall (r = 0.216, p<0.05), immediate recall (r = 0.189, p<0.01) and verbal fluency (r = 0.186, p<0.01). Similarly, Valenzuela et al. [31] demonstrated that the LEQ distinguished well between individuals with high and one with low lifespan activities and that variance-related discrimination was best in the mid-range of the proficiency distribution (maximum likelihood range estimate 0.08).

Regarding convergent validity, Valenzuela et al. [31] showed a moderate correlation between the LEQ and the Cognitive Activity Scale as a measure of leisure time activities (r = 0.41, p<0.0001).

There was poor evidence of structural validity and internal consistency of the LEQ. Item response theory analysis using a latent trait model with an option characteristic curve was performed with a sample of only 79 individuals and a questionnaire containing 42 items. Hence, it can be assumed that the analysis was underpowered [31]. Exploratory factor analysis identified 20 factors with eigenvalues >1.0, but the sample size cannot be considered sufficient for this analysis, therefore these factors were not considered as sub-scales for deriving Cronbach’s alphas. A poor Cronbach alpha was shown for the total scale (0.66). The Cronbach alphas of the sub-scales for the life stages ranged from 0.43 to 0.84, showing good alphas for the stage specific late-life sub-score and poor alphas for the stage specific young adulthood sub-score.

There was good evidence of the reliability of the LEQ with an intra-class test-retest correlation of r = 0.98.

Regarding responsiveness, Valenzuela et al. [31] found that cognitive decline (measured as difference between baseline and follow-up of a neuropsychological battery) was less pronounced in participants with higher LEQ scores over 18 months (r = 0.37, p = 0.003). Life-stage sub-scores showed a similar relationship with cognitive decline as the total LEQ score (young adulthood: r = 0.36, p = 0.005; mid-life: r = 0.35, p = 0.006; late-life: r = 0.22, p = 0.09).

### PCAS

There was fair evidence of content validity of the PCAS [32]. The items were selected from literature review and expert opinions. A pilot test was conducted to check the comprehensibility of the items. Several dimensions that may contribute to CR are not assessed in the questionnaire, such as specific leisure time activities, social activities, or bilingualism. However, these factors could be less relevant for the target population with low education and hence, may not necessarily be assessed for measuring CR in this population. Additionally, the PCAS does not assess CR-building activities throughout the life span, but only assesses cognitive abilities in the premorbid phase of people with dementia as well stable components such as education.

Regarding construct validity, the PCAS highly correlated with the Neuropsi total score (r = 0.73) in a group of 72 adults without cognitive impairment. However, no information on construct validity is available for people with cognitive impairment [32]. Regarding convergent validity, the PCAS correlated strongly with the Short Assessment of Health Literacy for Portuguese Speaking Adults as measure for premorbid intelligence (r = 0.82) in participants without cognitive impairment.

There was good evidence for structural validity of the PCAS. A two factor solution emerged for PCAS with good matrices indicators (Kaiser-Mayer-Olkin = 0.90; Barttlet’ sfericity, p <0.001) accounting for 50% of the total variance; factor 1 –advanced cognitive abilities (11 items) and factor 2 –basic reading and writing abilities (8 items) [32]. The internal consistency for the total scale was excellent with Cronbach’s alpha of 0.90 for the total scale, 0.85 for factor 1 and 0.87 for factor 2.

Inter-rater reliability was calculated from a sub-sample of the deceased participants. ICC was good with a value of 0.96 (95%CI = 0.92–0.99). There was no information on test-retest reliability, measurement error and responsiveness.

### RICE

There was good evidence of content validity of the RICE, since the items were chosen in collaboration with the target population and based on a literature review. However, detailed information on the conducted literature review was not reported. A pilot study was performed regarding relevance and comprehensibility of the items in the target population. The questionnaire assesses only activities during childhood and does not capture the whole life span, which limits the quality of the content validity.

Regarding convergent validity, a positive association between the RICE and years of education was observed (r = 0.32, p<0.001).

There was good evidence for the structural validity of the RICE. A 3-factor solution with reduced matrices indicators was reported (Kaiser–Meyer–Olkin = 0.81; Bartlett’s Test of Sphericity p<0.001). However, neither variance explained nor comparative fit index, root mean residuals, and root mean square error were reported, hindering a proper evaluation of the structural validity [33]. The internal consistency of the total scale was excellent and good to moderate for the sub-scales, respectively (Cronbach’s alpha = 0.79 for the total scale, Cronbach’s alpha = 0.72 for factor 1, Cronbach’s alpha = 0.76 for factor 2, Cronbach’s alpha = 0.69 for factor 3).

There was good evidence for the reliability of the RICE. Test-retest reliability as well as inter-rater reliability was good (ICC = 0.95, 95%CI = 0.90–0.97 and ICC = 0.99, 95%CI = 0.997–0.999, respectively). There was no information available regarding measurement error and responsiveness.

## Discussion

The methodological quality of studies on measurement properties of six questionnaires measuring CR was evaluated with the COSMIN taxonomy. Information on other measurement properties besides construct validity was scarce for the CRIq and the LEQ. However, construct validity was assessed thoroughly for the CRIq and almost half of the studies included all three dimensions of the CR hypothesis. Only one study on measurement properties was available for each, the PCAS and the RICE. However, since these questionnaires target a specific population, this is not surprising. Most information on different measurement properties was available for the CRS and the CRQ. Since the majority of the measurement properties per questionnaire were poorly or not assessed at all, a final conclusion regarding the methodological quality of the questionnaires cannot conclusively be drawn.

No study evaluated content validity directly, and we based our evaluation of content validity on information that was reported about the development of the questionnaire. However, information regarding the development of the questionnaires was often scarce. This does not mean that the content validity of the questionnaires is of bad quality, but rather indicates that content validity should be investigated in further research. Good content validity is a basic requirement for all other measurement properties. Although most authors report that the concept of CR is based on the theory of Stern et al. [2], there is no universal consensus on the definition of the hypothetical construct of CR and on the factors that should at least be considered to properly measure this construct. The included items of the questionnaires were often not evaluated with regard to their relevance regarding to the CR construct. Only two out of six questionnaires considered CR-building activities throughout various different life stages, namely the CRS and the LEQ [30,31]. Since CR can be enhanced in every life stage, and CR-building activities could differ in certain life-stages, it is important to assess these activities for the certain life stages separately to be able to measure CR accurately.

Cross-cultural validity was moderate for the Greek version of the CRIq [42] and the Portuguese version of the CRQ [53], while it was well performed for the Italian version of the CRS [58]. When adopting a questionnaire for another culture, an integral and precise translation by an expert including forward and backward translation, item revision and a pretest of the translated version in the target population is required [26,67]. A mere translation is not sufficient, since CR-building activities could differ across cultures, as shown by the two questionnaires that target a more specific population [32,33].

Structural validity was often not assessed or not assessed properly (as in the case of CRS). Still, several studies on the CRS and one on the CRIq reported the internal consistency without evaluating structural validity [24, 30, 58,60], or even when unidimensionality was questionable [63].

Concerning construct validity, different target populations were considered and the cognitive measures were heterogeneous across studies. There is no consensus on how CR construct and convergent validity should be assessed. There is a lack of information whether in diseased populations cognitive decline is faster in people with high CR when compared to people with low CR after the onset of cognitive deficits. Longitutinal data are nessecary to test this hypothesis and would strengthen the information on construct validity of existing CR questionnaires. It is difficult to define at which time point or at which level of cognitive deficits neuropathology is too severe and people with high CR will have a rapid cognitive decline when compared to people with low CR. In our opinion, the best way to evaluate the construct validity is to include also neuropathology measures. However, this is often not possible due to financial or personal contraints. When evaluating contruct validity, the study population needs to be selected carefully and the expected direction and strength of associations with CR measures and cognitive outcomes needs to be stated beforehand, which was not always the case in the mentioned studies.

The CRIq and the CRQ seemed to be valid for diseased populations, but evidence is limited for the healthy elderly population. On the other hand, the CRS seems to be valid for the healthy elderly population. Information regarding the construct validity of the LEQ, the PCAS, and the RICE is too scarce and no conclusions can be drawn.

In general, responsiveness shall detect changes in the measured construct. However, when CR is the construct of interest, it is important that changes in the cognitive outcome measures can be detected with regards to the specific CR score. The measured CR could be used as a tool to predict cognitive outcomes and if necessary, appropriate interventions could be used regarding the CR score in order to optimize these outcomes. Additionally, if the identification of risk groups for cognitive decline is of importance, information on responsiveness of the questionnaires is crucial. However, evidence on the responsiveness of the questionnaires is limited and only available for the CRIq [43], the CRQ [57], and the LEQ [31], with high methodological quality only observed for the LEQ. As stated for construct validity, good methodological studies using longitudinal data are necessary to evaluate responsiveness of CR questionnaires.

Whether a CR questionnaire is able to detect people with low and high CR using a defined cut-off was not examined by any of the studies and should be an aim for further investigations. Evidence on cut-points able to categorize people in having low and high CR is nessecary especially in clinical practice for interpreting the score of a specific patient.

The main strength of our systematic review is the inclusion of all identified studies irrespective of language or population examined and with no time restriction, which extends the external validity of this review. Additionally, we applied the COSMIN checklist for assessing the risk of bias in the studies and for the synthesis of the results, a checklist which is consensus-based and standardized. However, there are also a few limitations. First, for many of the questionnaires there was an inadequate or selective reporting of measurement properties in the reviewed literature, which makes an appropriate qualification of the measures difficult. Second, due to a relatively recent development of the questionnaires under examination, this review was based on only few studies for each CR questionnaire. This limited number of studies led to many measurement properties being measured in only one study. Third, publication bias may have affected the results presented for the included studies. Fourth, we rated the quality of different brain pathology measures equally, since to some extent all brain pathology measures could be correlated with the actual underlying pathology.

Generally, there is a lack of sufficiently good information about the measurement properties of questionnaires measuring CR. However, since the instruments are all relatively new, this is a plausible situation, requiring further research efforts in order to refine the measurement of CR. In that respect, future research needs to evaluate the psychometric properties of the identified questionnaires using appropriate methods at the design stage and for the analysis stage of data processing. The COSMIN checklist can be used for planning such a study on measurement properties. We recommend evaluating especially the content validity, structural validity, and responsiveness of the identified questionnaires. Developing a new questionnaire to measure CR is not considered an important priority at this time. We rather recommend evaluating the recently developed questionnaires to be able to measure CR appropriately in epidemiological and experimental studies.

A final recommendation for one of the questionnaires cannot be drawn, because many measurement properties were not sufficiently reported by the corresponding studies, thus, preventing a clear conclusion. However, in our opinion the LEQ and the CRS represent promising questionnaires for measuring CR. The LEQ is a long questionnaire containing many different important CR proxies while the CRS is a relatively short questionnaire that could be introduced in large epidemiological studies. The CRS represents a measure of CR beyond education and occupational status (information that is anyway collected in epidemiological studies) and is able to give a more complete picture of CR in these studies.

The application of various CR questionnaires in larger epidemiological samples might provide further information about the large scale validity of the questionnaire and its appropriateness for reflecting differences between various subject groups differing in disease, age, and so forth. On the other hand, experimental studies focusing on the application of a certain paradigm to measure cognitive and other skills might be conducted to understand the specific background of a proxy assumed to reflect certain aspects of CR. CR questionnaires can be also useful in clinical settings to identify people at risk for developing cognitive impairment. Interventions to enhance CR could be promoted to people with low CR in order to postpone cognitive deficits in diseases such as AD, MS, or due to aging. On the other hand, people with high CR, but cognitive performance within lower normal ranges should be examined further, as this already might be a first clinical sign of cognitive decline in those people [24]. Beside the clinical settings and taking into account that CR enhancing activities can be promoted at every life stage, the measurement of CR could be extended to various different groups and situations, e.g. healthy retired citizens, people with emotional disturbances, immigrants, and adolescents with social risk factors. The present review might be of help to specify the corresponding measures that enable a well-informed investigation of CR of these people.

## Supporting information

S1 File Supporting information PRISMA. (PDF)

S2 File Supporting materials–Questionnaires description. (PDF)
