# Supplementary material for: Measuring Cognitive Reserve (CR) – A systematic review of measurement properties of CR questionnaires for the adult population
Source: PLoS One. 2019 Aug 7;14(8):e0219851. doi: 10.1371/journal.pone.0219851 (PMC6685632; doi:10.1371/journal.pone.0219851)
Supplement: S2 File — (PDF) [file pone.0219851.s002.pdf]

S2 – File

Description of scales

CRIq

The CRIq was developed (Nucci, Mapelli et al. 2012) and validated initially in Italian (Puccioni and Vallesi 2012, Milanini, Ciccarelli et al. 2016, Mondini, Madella et al. 2016, Nunnari, De Cola et al. 2016, Amodio, Montagnese et al. 2017, Arcara, Mondini et al. 2017, Volpi, Pagni et al. 2017, Ciccarelli, Monaco et al. 2018, Fenu, Lorefice et al. 2018). A Greek translation with fair cross-cultural validation has been conducted (Maiovis, Ioannidis et al. 2016) and further used (Maiovis, Ioannidis et al. 2018). Additionally, there are versions available in English, French, German, Spanish, Portuguese, Catalan, Czech, Dutch and Latvian (http://www.cognitivereserveindex.org/), although no information on cross-cultural validation is available. The CRIq is a questionnaire that consists of 24 items divided in three subscales: education, working activity and leisure time with dichotomous answers. A total score is calculated combining the three sub-scores. The raw score is normalized to a score with mean of 100 and standard deviation (SD) of 15. The CRIq can be filled in by the participant or a participant's caregiver, relative, or friend within 15 minutes.

CRQ

The CRQ was developed (Rami, Valls-Pedret et al. 2011) and validated in Spanish (Lopez-Higes, Rubio-Valdehita et al. 2013, López-Higes 2014, Pedrero-Perez, Rojo-Mota et al. 2014, Harris, Fernandez Suarez et al. 2015, Ferreira, Bartres-Faz et al. 2016, Vasquez-Amezquita 2016, Bartres-Faz, Cattaneo et al. 2018, Lopez-Higes, Martin-Aragoneses et al. 2018, Wikee and Martella 2018). A Portuguese translation with fair cross-cultural validation was performed (Sobral, Pestana et al. 2014, Sobral, Pestana et al. 2015). No versions in further languages were found. The CRQ is a very short questionnaire composed of 8 items on a 3 and 6-point Likert scale. A total score, ranging from 0 to 25, is calculated as the sum of the scores from each item. Hence, the items are equally weighted. Higher scores are indicative of higher CR. The CRQ can be filled in by the participant self or a relative, friend, or caregiver. Poor evidence on content validity was available for the CRQ (Rami, Valls-Pedret et al. 2011). The authors reported that the items were based on the CR theory of Stern (Stern 2009), but did not provide any information regarding the development, pilot testing, relevance, comprehensibility and comprehensiveness of the items. However, the questionnaire includes the important proxy indicators of the CR construct.

CRS

The CRS was developed (Leon, Garcia et al. 2011) and validated in Spanish (Leon, Garcia-Garcia et al. 2014, Leon, Garcia-Garcia et al. 2016, Leon-Estrada, Garcia-Garcia et al. 2017, Roldan-Tapia, Canovas et al. 2017, Cancino, Rehbein-Felmer et al. 2018). An Italian translation with good cross-cultural validation has been performed (Altieri, Siciliano et al. 2018), and a non-validated English version is available under http://www2.ual.es/CognitiveReserveScale/en/escala-de-reserva-cognitiva-erc/. To our knowledge, no versions in other languages are available. The CRS is a 24-items self-administered questionnaire. It is divided into four categories: daily activities, training information, hobbies, and social life with answers on a 5-point Likert scale. It ranges from 0 (never) to 4 (three or more times per week). It contains three life stages: young adulthood (18–35 years), adulthood (36–64 years) and late adulthood (≥ 65 years). Participants are required to respond to the life-stages subscales according to their age. The total score ranges from 0 to 96, where higher scores indicate more frequent participation in activities and higher levels of CR in each life stage. A total CRS score is obtained by summing up the mean score on each item.

LEQ

The LEQ was initially developed (Valenzuela and Sachdev 2007) and validated in English (Opdebeeck, Nelis et al. 2015, Lavrencic, Kurylowicz et al. 2016, Hindle, Martin-Forbes et al. 2017). A further German version has been validated in the DELCODE study. However, since only a conference abstract without detailed information on the German validation is available, we excluded this study. The LEQ consists of 42 questions with answers on a 6-point Likert scale and open questions for frequency and intensity of participation on different activities. The LEQ consists of two dimensions: specific and non-specific mental activity assessed in three life-stages: young adulthood (13-30 years); midlife (30-65 years or until retirement) and late-life (65 or from retirement onwards). The total LEQ score is the sum of the three life-stage scores and higher scores are indicative of higher CR. Since the LEQ is a very thorough questionnaire, a very broad range of CR proxies are included in the questionnaire. Additionally, different life stages are examined in the LEQ with different items for each life stage, contributing to the quality of content validity.

PCAS

The PCAS was originally developed and validated in Portuguese (Apolinario, Brucki et al. 2013). To our knowledge, the scale is not available in other languages. This instrument was developed to estimate premorbid cognitive abilities in low-educated populations. It consists of 19 items addressing educational attainment (5 points), major lifetime occupation (4 points), reading abilities (4 points), writing abilities (4 points), use of technology (3 points), abilities to search for specific information (4

points), reading habits (4 points), and calculation abilities (2 points). The resulting instrument provides scores in a range from 0 to 30.

RICE

The RICE was originally developed and validated in English (Minogue, Delbaere et al. 2018), with Australian Aboriginal people as target population. The RICE is not available in other languages. This scale consists of 21 questions about exposure to participation in traditional Aboriginal cultural activities, community events, physical activity, reading and playing games. Each question has a 5-point Likert scale answer (1=never to 5=a lot). A total score ranges from 19 to 81, with lower scores indicating lower CR.

Altieri, M., M. Siciliano, S. Pappacena, M. D. Roldan-Tapia, L. Trojano and G. Santangelo (2018). "Psychometric properties of the Italian version of the Cognitive Reserve Scale (I-CRS)." Neurol Sci **39**(8): 1383-1390.

Amodio, P., S. Montagnese, G. Spinelli, S. Schiff and D. Mapelli (2017). "Cognitive reserve is a resilience factor for cognitive dysfunction in hepatic encephalopathy." Metab Brain Dis **32**(4): 1287-1293.

Apolinario, D., S. M. Brucki, R. E. Ferretti, J. M. Farfel, R. M. Magaldi, A. L. Busse and W. Jacob-Filho (2013). "Estimating premorbid cognitive abilities in low-educated populations." PLoS One **8**(3): e60084.

Arcara, G., S. Mondini, A. Bisso, K. Palmer, F. Meneghello and C. Semenza (2017). "The Relationship between Cognitive Reserve and Math Abilities." Front Aging Neurosci **9**: 429.

Bartres-Faz, D., G. Cattaneo, J. Solana, J. M. Tormos and A. Pascual-Leone (2018). "Meaning in life: resilience beyond reserve." Alzheimers Res Ther **10**(1): 47.

Cancino, M., L. Rehbein-Felmer and M. S. Ortiz (2018). "[Cognitive reserve, depression and social support. Analysis of 206 older adults]." Rev Med Chil **146**(3): 315-322.

Ciccarelli, N., M. R. L. Monaco, D. Fusco, D. L. Vetrano, G. Zuccala, R. Bernabei, V. Brandi, M. S. Pisciotta and M. C. Silveri (2018). "The role of cognitive reserve in cognitive aging: what we can learn from Parkinson's disease." Aging Clin Exp Res **30**(7): 877-880.

Fenu, G., L. Lorefice, M. Arru, V. Sechi, L. Loi, F. Contu, F. Cabras, G. Coghe, J. Frau, M. Fronza, G. Sbrescia, V. Lai, M. Boi, S. Mallus, S. Murru, A. Porcu, M. A. Barracciu, M. G. Marrosu and E. Cocco (2018). "Cognition in multiple sclerosis: Between cognitive reserve and brain volume." J Neurol Sci **386**: 19-22.

Ferreira, D., D. Bartres-Faz, L. Nygren, L. J. Rundkvist, Y. Molina, A. Machado, C. Junque, J. Barroso and E. Westman (2016). "Different reserve proxies confer overlapping and unique endurance to cortical thinning in healthy middle-aged adults." Behav Brain Res **311**: 375-383.

Harris, P., M. Fernandez Suarez, E. I. Surace, P. Chrem Mendez, M. E. Martin, M. F. Clarens, F. Tapajoz, M. J. Russo, J. Campos, S. M. Guinjoan, G. Sevlever and R. F. Allegri (2015). "Cognitive reserve and Abeta1-42 in mild cognitive impairment (Argentina-Alzheimer's Disease Neuroimaging Initiative)." Neuropsychiatr Dis Treat **11**: 2599-2604.

Hindle, J. V., P. A. Martin-Forbes, A. Martyr, A. J. M. Bastable, K. L. Pye, V. C. Mueller Gathercole, E. M. Thomas and L. Clare (2017). "The effects of lifelong cognitive lifestyle on executive function in older people with Parkinson's disease." Int J Geriatr Psychiatry **32**(12): e157-e165.

Lavrencic, L. M., L. Kurylowicz, M. J. Valenzuela, O. F. Churches and H. A. Keage (2016). "Social cognition is not associated with cognitive reserve in older adults." Neuropsychol Dev Cogn B Aging Neuropsychol Cogn **23**(1): 61-77.
Leon-Estrada, I., J. Garcia-Garcia and L. Roldan-Tapia (2017). "[Cognitive Reserve Scale: testing the theoretical model and norms]." Rev Neurol **64**(1): 7-16.
Leon, I., J. Garcia-Garcia and L. Roldan-Tapia (2014). "Estimating Cognitive Reserve in Healthy Adults Using the Cognitive Reserve Scale." Plos One **9**(7): e102632.
Leon, I., J. Garcia-Garcia and L. Roldan-Tapia (2016). "Cognitive Reserve Scale and ageing." Anales De Psicologia **32**(1): 218-223.
Leon, I., J. Garcia and L. Roldan-Tapia (2011). "[Development of the scale of cognitive reserve in Spanish population: a pilot study]." Rev Neurol **52**(11): 653-660.
Lopez-Higes, R., M. T. Martin-Aragoneses, S. Rubio-Valdehita, M. L. Delgado-Losada, P. Montejo, M. Montenegro, J. M. Prados, J. de Frutos-Lucas and D. Lopez-Sanz (2018). "Efficacy of Cognitive Training in Older Adults with and without Subjective Cognitive Decline Is Associated with Inhibition Efficiency and Working Memory Span, Not with Cognitive Reserve." Front Aging Neurosci **10**: 23.
Lopez-Higes, R., S. Rubio-Valdehita, J. M. Prados and M. Galindo (2013). "[Cognitive reserve and linguistic skills in healthy elderly persons]." Rev Neurol **57**(3): 97-102.
López-Higes, R. R.-V., S. (2014). "[Variability in grammatical comprehension in healthy older adults: Differences according to cognitive reserve]." Revista de Logopedia, Foniatría y Audiología **34**(2): 51-59.
Maiovis, P., P. Ioannidis, G. Gerasimou, A. Gotzamani-Psarrakou and D. Karacostas (2018). "Cognitive Reserve Hypothesis in Frontotemporal Dementia: Evidence from a Brain SPECT Study in a Series of Greek Frontotemporal Dementia Patients." Neurodegener Dis **18**(2-3): 69-73.
Maiovis, P., P. Ioannidis, M. Nucci, A. Gotzamani-Psarrakou and D. Karacostas (2016). "Adaptation of the Cognitive Reserve Index Questionnaire (CRIq) for the Greek population." Neurol Sci **37**(4): 633-636.
Milanini, B., N. Ciccarelli, M. Fabbiani, S. Limiti, P. Grima, B. Rossetti, E. Visconti, E. Tamburrini, R. Cauda and S. Di Giambenedetto (2016). "Cognitive reserve and neuropsychological functioning in older HIV-infected people." J Neurovirol **22**(5): 575-583.
Minogue, C., K. Delbaere, K. Radford, T. Broe, W. S. Forder and S. Lah (2018). "Development and initial validation of the Retrospective Indigenous Childhood Enrichment scale (RICE)." Int Psychogeriatr **30**(4): 519-526.
Mondini, S., I. Madella, A. Zangrossi, A. Bigolin, C. Tomasi, M. Michieletto, D. Villani, G. Di Giovanni and D. Mapelli (2016). "Cognitive Reserve in Dementia: Implications for Cognitive Training." Front Aging Neurosci **8**: 84.
Nucci, M., D. Mapelli and S. Mondini (2012). "Cognitive Reserve Index questionnaire (CRIq): a new instrument for measuring cognitive reserve." Aging Clin Exp Res **24**(3): 218-226.
Nunnari, D., M. C. De Cola, A. Costa, C. Rifici, P. Bramanti and S. Marino (2016). "Exploring cognitive reserve in multiple sclerosis: New findings from a cross-sectional study." J Clin Exp Neuropsychol **38**(10): 1158-1167.
Opdebeeck, C., S. M. Nelis, C. Quinn and L. Clare (2015). "How does cognitive reserve impact on the relationships between mood, rumination, and cognitive function in later life?" Aging Ment Health **19**(8): 705-712.
Pedrero-Perez, E. J., G. Rojo-Mota, J. M. Ruiz-Sanchez de Leon, L. M. Fernandez-Mendez, S. Morales-Alonso and A. Prieto-Hidalgo (2014). "[Cognitive reserve in substance addicts in treatment: relation to cognitive performance and activities of daily living]." Rev Neurol **59**(11): 481-489.
Puccioni, O. and A. Vallesi (2012). "High cognitive reserve is associated with a reduced age-related deficit in spatial conflict resolution." Front Hum Neurosci **6**: 327.
Rami, L., C. Valls-Pedret, D. Bartres-Faz, C. Caprile, C. Sole-Padulles, M. Castellvi, J. Olives, B. Bosch and J. L. Molinuevo (2011). "[Cognitive reserve questionnaire. Scores obtained in a healthy elderly population and in one with Alzheimer's disease]." Rev Neurol **52**(4): 195-201.
Roldan-Tapia, M. D., R. Canovas, I. Leon and J. Garcia-Garcia (2017). "Cognitive Vulnerability in Aging May Be Modulated by Education and Reserve in Healthy People." Front Aging Neurosci **9**: 340.

Sobral, M., M. H. Pestana and C. Paul (2014). "Measures of cognitive reserve in Alzheimer's disease." Trends Psychiatry Psychother **36**(3): 160-168.
Sobral, M., M. H. Pestana and C. Paul (2015). "Cognitive reserve and the severity of Alzheimer's disease." Arq Neuropsiquiatr **73**(6): 480-486.
Stern, Y. (2009). "Cognitive reserve." Neuropsychologia **47**(10): 2015-2028.
Valenzuela, M. J. and P. Sachdev (2007). "Assessment of complex mental activity across the lifespan: development of the Lifetime of Experiences Questionnaire (LEQ)." Psychol Med **37**(7): 1015-1025.
Vasquez-Amezquita, M. (2016). "Predictors of cognitive reserve in a group of elderly." Revista Chilena De Neuropsicologia **11**(1): 5-11.
Volpi, L., C. Pagni, C. Radicchi, S. Cintoli, M. Miccoli, U. Bonuccelli and G. Tognoni (2017). "Detecting cognitive impairment at the early stages: The challenge of first line assessment." J Neurol Sci **377**: 12-18.
Wikee, G. and D. Martella (2018). "[Physical activity and cognitive reserve as protective factors for attentional functioning in older people]." Rev Med Chil **146**(5): 570-577.
